# Alignment of adherence and risk for HIV acquisition in a demonstration project of pre-exposure prophylaxis among HIV serodiscordant couples in Kenya and Uganda: a prospective analysis of prevention-effective adherence

**DOI:** 10.7448/IAS.20.1.21842

**Published:** 2017-07-25

**Authors:** Jessica E. Haberer, Lara Kidoguchi, Renee Heffron, Nelly Mugo, Elizabeth Bukusi, Elly Katabira, Stephen Asiimwe, Katherine K. Thomas, Connie Celum, Jared M. Baeten

**Affiliations:** ^a^ Massachusetts General Hospital Global Health, Boston, MA, USA; ^b^ Department of Medicine, Harvard Medical School, Boston, MA, USA; ^c^ Department of Global Health, University of Washington, Seattle, WA, USA; ^d^ Kenya Medical Research Institute, Thika, Kenya; ^e^ Kenya Medical Research Institute, Kisumu, Kenya; ^f^ Infectious Disease Institute, Kampala, Uganda; ^g^ Kabwohe Clinical Research Centre, Kabwohe, Uganda

**Keywords:** prevention-effective adherence, pre-exposure prophylaxis, PrEP

## Abstract

**Introduction:** Adherence is essential for pre-exposure prophylaxis (PrEP) to protect against HIV acquisition, but PrEP use need not be life-long. PrEP is most efficient when its use is aligned with periods of risk – a concept termed prevention-effective adherence. The objective of this paper is to describe prevention-effective adherence and predictors of adherence within an open-label delivery project of integrated PrEP and antiretroviral therapy (ART) among HIV serodiscordant couples in Kenya and Uganda (the Partners Demonstration Project).

**Methods**: We offered PrEP to HIV-uninfected participants until the partner living with HIV had taken ART for ≥6 months (a strategy known as “PrEP as a bridge to ART”). The level of adherence sufficient to protect against HIV was estimated in two ways: ≥4 and ≥6 doses/week (per electronic monitoring). Risk for HIV acquisition was considered *high* if the couple reported sex with <100% condom use before six months of ART, *low* if they reported sex but had 100% condom use and/or six months of ART and *very low* if no sex was reported. We assessed prevention-effective adherence by cross-tabulating PrEP use with HIV risk and used multivariable regression models to assess predictors of ≥4 and ≥6 doses/week.

**Results**: A total of 985 HIV-uninfected participants initiated PrEP; 67% were male, median age was twenty-nine years, and 67% reported condomless sex in the month before enrolment. An average of ≥4 doses and ≥6 doses/week were taken in 81% and 67% of participant-visits, respectively. Adherence sufficient to protect against HIV acquisition was achieved in 75–88% of participant-visits with high HIV risk. The strongest predictor of achieving sufficient adherence was reporting sex with the study partner who was living with HIV; other statistically significant predictors included no concerns about daily PrEP, pregnancy or pregnancy intention, females aged >25 years, older male partners and desire for relationship success. Predictors of not achieving sufficient adherence were no longer being a couple, delayed PrEP initiation, >6 months  of follow-up, ART use >6 months  by the partner living with HIV and problem alcohol use.

**Conclusions:** Over three-quarters of participant-visits by HIV-uninfected partners in serodiscordant couples achieved prevention-effective adherence with PrEP. Greater adherence was observed during months with HIV risk and the strongest predictor of achieving sufficient adherence was sexual activity.

## Introduction

Antiretroviral-based pre-exposure prophylaxis (PrEP) is a highly efficacious means for prevention of HIV acquisition; however, its efficacy depends greatly on adherence, as was demonstrated in numerous clinical trials [1]. At the individual level, high adherence to PrEP has been associated with ≥90% protection from HIV, as assessed by detection of the medication in blood samples [2,3]. Pharmacologic modelling has associated dosing ≥4 tablets/week with 96% HIV risk reduction in studies of men who have sex with men (MSM) [4], while *ex vivo* viral challenge studies of vaginal tissue suggest ≥6 doses/week may be necessary for HIV protection in women [5].

Open-label demonstration studies (i.e. studies of methods for PrEP delivery) to date have indicated high levels of adherence among heterosexual populations and MSM. The Partners Demonstration Project recruited high-risk heterosexual HIV serodiscordant couples in Kenya and Uganda and found detectable plasma tenofovir levels in 85% of a random sample of participants [6]. The Demo Project recruited MSM in the USA and found dried blood spot tenofovir levels consistent with ≥4 doses/week in ≥80% of participants [7]. HIV incidence in both demonstration projects was low (0.2 and 0.43/100 person-years, respectively). A smaller study (HPTN 067/ADAPT) similarly found high adherence among young women in South Africa (76% for daily dosing) [8]. These findings are highly encouraging and suggest that at-risk individuals can adhere to PrEP when offered in non-clinical trial settings and with the knowledge that PrEP is effective when taken.

PrEP adherence is necessary for HIV protection, but PrEP adherence need not be life-long; use should be aligned with periods of HIV risk. A detailed understanding of adherence and its alignment with risk behaviours associated with HIV acquisition is critical for both individual and public health benefits. Low PrEP adherence or non-use in the setting of risk may lead to HIV acquisition, but PrEP non-use in the absence of risk is both acceptable and arguably rational. Conversely, high adherence when HIV risk is present is the ideal, but high adherence in the absence of risk is undesirable because of its inefficiency, patient burden, potential for side effects (although rare) and unnecessary cost. Efficient use of PrEP is a major consideration for its roll-out in both low- and well-resourced settings, given multiple competing priorities among other treatment and prevention efforts [9]. Adherence, therefore, should be considered in the context of HIV risk – a concept that has been termed as prevention-effective adherence [10].

Within an open-label delivery project of integrated PrEP and antiretroviral therapy (ART) among HIV-uninfected members of HIV serodiscordant couples in Kenya and Uganda (the Partners Demonstration Project), we prospectively measured adherence to PrEP, as well as sexual behaviour and other markers of HIV risk. We then used this data to describe prevention-effective adherence and predictors of adherence. This paper thus presents the first analysis of prevention-effective adherence using data on time-limited PrEP use by HIV-uninfected persons with a known partner living with HIV.

## Methods

### Partners Demonstration Project

The Partners Demonstration Project was a prospective, open-label, implementation science-driven study of ART and PrEP for HIV prevention among high-risk heterosexual HIV serodiscordant couples in Kenya and Uganda [6]. The overall goal was to evaluate a scalable, integrated and pragmatic delivery approach for ART and time-limited PrEP, with targeted counselling, brief adherence promotion and frequency of follow-up designed to reflect approaches suitable for public health delivery in resource-limited settings.

Beginning in November 2012, couples were recruited using community outreach methods by four clinical care and research sites in Kenya (Kisumu and Thika) and Uganda (Kabwohe and Kampala). Eligible couples were ≥18 years of age, sexually active and intending to remain as a couple for at least one year. A validated, empiric risk score involving age, number of children, male circumcision, marital/cohabitation status, sexual activity and viral load was used during screening; couples with a score ≥5 of 13 points were eligible for enrolment. In prior studies of HIV serodiscordant couples, a score ≥5 was associated with an HIV incidence in excess of 3–4% per year [11]. A sample size of 1000 was chosen to provide a robust evaluation of the integrated ART and PrEP delivery strategy, across a diversity of clinical research sites.

### Study procedures

At enrolment, couples were counselled on the HIV prevention benefits of immediate PrEP and ART. HIV-uninfected partners were offered PrEP (combination emtricitabine 200 mg/tenofovir disoproxil fumarate 300 mg once daily), which was provided at study sites as PrEP had extremely limited availability otherwise in Kenya and Uganda during the study period. PrEP was recommended for use until the partner living with HIV had been on ART for >6 months, permitting time to achieve viral suppression, with PrEP discontinuation encouraged thereafter (a strategy characterized as “PrEP as a bridge to ART”). Partners living with HIV were referred to receive ART either onsite or at a public health clinic of their choice, following national guidelines that initially required a CD4 count <350 cells/µl or symptomatically advanced HIV disease. In 2014, Kenyan and Ugandan ART guidelines were expanded to recommend use for all persons with HIV-negative partners regardless of clinical indications. ART use was obtained through self-report. For couples in which the partner living with HIV delayed or declined ART, the PrEP “bridge” period was extended until ART was initiated and sustained for greater than equal to six months. PrEP use could overlap with ART use for >six months, if desired by the HIV-uninfected partner and if the clinician felt there was rationale to continue. Couples returned for follow-up visits at one month after enrolment and then quarterly for up to twenty-four months. Visits included HIV testing for HIV-uninfected partners, PrEP dispensing, and brief adherence and HIV risk reduction counselling. Counselling messages were consistent with US Centers for Disease Control and Prevention guidance [12], including daily PrEP use [13]. Participants also completed interviewer-administered questionnaires at study visits (questionnaire data presented here reflect the HIV-uninfected partner unless otherwise stated). Sexual behaviour was reported over the prior month. Heavy alcohol use was defined as a positive Rapid Alcohol Problems Screen [14]. Depression was assessed by the Hopkins Checklist, as suggested by a score of >1.75 [15]. Belief in PrEP efficacy was assessed by standardized questionnaire after counselling on the clinical trial efficacy data. Social support was assessed by the Duke-UNC Social Support Scale [16]. Perceived HIV stigma was evaluated with a modified Berger scale [17,18]. Partnership discordance was based on three questions derived through qualitative research within the Partners PrEP Study [19], and relationship satisfaction was assessed through the dyadic adjustment scale [20]. Follow-up for the study concluded in June 2016.

### PrEP adherence

PrEP was distributed in bottles with medication event monitoring system (MEMS, WestRock, Switzerland) caps to electronically capture a date-and-time stamp for each pill bottle opening. These electronic data were downloaded at each visit and used only for measurement of adherence; they were not incorporated into adherence counselling.

Data in this analysis reflect the first twelve months of follow-up after PrEP initiation for each participant. Adherence was calculated as the number of openings divided by the number of expected openings during the total number of days for which PrEP had been dispensed and a drug hold was not in effect in the month prior to each participant-visit. Openings by study staff were not counted (e.g. openings only to switch medication bottles). Participant-visits were excluded if the participant missed the visit, had missing adherence data (e.g. a broken or lost device), protocol-defined PrEP holds (e.g. due to an adverse event) or had adherence >120%, likely reflecting device error or unexpected device use (e.g. opening the pill bottle multiple times without removing a dose). In a sensitivity analysis, openings >2 per day were not counted.

To examine prevention-effective adherence (i.e. adherence sufficient for protection against HIV acquisition aligned with periods of HIV risk), we made the following classifications using data from the month prior to each study visit:

- *Risk for HIV acquisition*: Risk for HIV was considered *high* if the couple reported sex with <100% condom use prior to six months’ duration of ART use by the study partner who was living with HIV. Risk was considered *low* if the couple reported sex but did not meet the definition of high risk (i.e. they had 100% reported condom use and/or six months of ART use), and risk was considered *very low* if no sex was reported (regardless of ART use). Data on sex with outside partners were not included in this analysis, as the HIV status of those partners was unknown. Because previously reported data [21] indicate potential risk due to these outside partnerships and self-reported sexual behaviour and ART use could not be confirmed, all participants were considered to have some degree of HIV risk.
- *Sufficient adherence*: The level of adherence sufficient for prevention of HIV acquisition depends on numerous factors, including drug metabolism and tissue of exposure. Clinical data on rectal exposure suggest that an average of ≥4 doses/week provides potentially ≥96% protection against HIV infection [4], whereas pharmacologic modelling on vaginal exposure suggest that an average of ≥6 doses/week is needed to achieve similar levels of protection [5]. No data are available on penile exposure. For the purposes of this analysis, both ≥4 doses/week and ≥6 doses/week on average were considered as estimates of sufficient adherence.

### Statistical analysis

Prevention-effective adherence was examined via cross-tabulation of participant-visits classified into the three categories of risk for HIV acquisition versus sufficient adherence as described above. Comparisons of proportions with sufficient adherence among risk categories were made by Fisher’s exact test. Subgroup assessments were made for young adults (age <25), women and young women, based on adherence challenges seen in these populations in other studies [22–24], and compared to the remainder of the cohort using the Wilcoxon rank sum test. Factors associated with sufficient adherence were evaluated using univariable and multivariable generalized estimating equation models with logistic link and robust standard errors to account for repeated measures, adjusting for study site. Separate models were developed for each estimate of sufficient adherence. Time-varying variables were measured concurrently with adherence behaviour at each study visit unless indicated otherwise. *A priori* interactions were assessed between gender and the following variables based on prior associations in the literature [25–29]: pregnancy intentions, alcohol use, depression, relationship satisfaction and abuse. Interactions were also assessed between gender and age. ART use >6 months  by the partner living with HIV was included because continued PrEP use in this scenario may have reflected possible impressions by the HIV-uninfected partner of poor ART adherence or of otherwise unreported outside partnerships; ART adherence was not measured directly. The multivariable model was adjusted for variables for which the *p*-value on univariable analysis was ≤0.10 (Wald test for continuous and binary measures; Type 3 score test for >2 categorical measures). All statistical analyses were conducted with SAS 9.4 (Cary, North Carolina, USA).

### Ethics statement

The study protocol was approved by the University of Washington Human Subjects Division (STUDY00001674) and ethics review committees at each study site (Kabwohe: UNCST HS1410, NARC 135; Kampala: UNCST HS1289, NARC 126; Kisumu: KEMRI SSC NO 2441; Thika: KEMRI P286/05/2012). Participants provided written informed consent.

## Results

### Study participants

A total of 1013 HIV serodiscordant couples were enrolled in the Partners Demonstration Project, and 985 (97%) of the HIV-uninfected partners initiated PrEP (Table 1). Less than 1% of participants initiated PrEP greater than one month into the study. The majority of HIV-uninfected partners were male (67%) and the median age was twenty-nine years. More than half of couples (56%) had no children together, 67% had condomless sex in the month prior to enrolment and the median viral load of partners living with HIV was 4.6 log_10_ copies/ml. Four participants seroconverted after enrolment into the Partners Demonstration Project.

**Table 1.** Enrolment characteristics for the HIV-uninfected participants who initiated PrEP (*N* = 985)

Individual characteristics	*N* (%) or median (IQR)
Female	329 (33%)
Age (years)	29 (26–36)
Education (years)	8 (6–12)
Circumcised (males only)	440 (67%)
“No concerns about daily PrEP”	878 (89%)
Risk of getting HIV is “moderate” or “high”	315 (32%)
“PrEP makes sex completely safe”	434 (44%)
Unprotected sex with study partner in past month^1^	638 (67%)
Unprotected sex with non-study partner in past month^1^	60 (6%)
Currently trying to get pregnant^2^	63 (7%)
Problem alcohol use	199 (20%)
Depression	101 (10%)
As much social support as desired	622 (63%)
Perceived HIV stigma is “moderate” or “high”	135 (14%)
**Partnership characteristics**	
Married to study partner	931 (95%)
Living with study partner	956 (97%)
In polygamous marriage	138 (14%)
Male is ≥five years older than the female	432 (44%)
Number of children with study partner	0 (0–2)
Has no children with study partner	554 (56%)
Aware of HIV discordance before enrolment	778 (79%)
Abuse reported in the partnership	7 (<1%)
Effect of discordance on relationship is “moderate” or “high”	536 (54%)
Relationship satisfaction^3^	11 (8–13)
Wants “very much” or “desperately” for relationship to succeed	862 (88%)
CD4 count of partner living with HIV	437 (271–640)
HIV RNA of partner living with HIV (log_10_)	4.6 (3.9–5.0)

IQR: Interquartile range. ^1^Denominator is 953, as 32 participants reported no sex in the past month. ^2^Denominator indicates couples where neither member was already pregnant at enrolment (*N* = 850). Pregnancy was an enrolment exclusion criterion only for the HIV-uninfected member of the couple. ^3^On a scale of 0–16, with higher indicating more satisfaction.

Participant retention at each study visit ranged from 86% to 91%. Twenty-three participants had no follow-up during the analysis period, leaving 962 participants with 4766 participant-visits of follow-up. Participant-visits were then excluded from the analysis for the following reasons: 790 due to missed visits or study protocol-related PrEP holds, 561 due to missing electronic adherence data, and 23 due to electronic adherence >120%. The remaining 3392 participant-visits of follow-up among 901 participants provided adherence data for analysis. An additional 107 participant-visits were missing data on HIV risk (sexual behaviour and/or ART use by the partner living with HIV), leaving 3285 (69%) participant-visits among 886 (92%) participants for assessment of prevention-effective adherence.

### Prevention-effective adherence

As shown in Table 2, participants’ overall median PrEP adherence was 88% (interquartile range [IQR] 64–99%) with a mean of 77% (standard deviation 28%); nearly identical values were seen when excluding >2 pill bottle openings per day. Compared to the remainder of the cohort, adherence was lower for young adults aged less than twenty-five years (83% [IQR 58–96%]; *p* = 0.06), higher for all women (91% [IQR 66–99%]; *p* = 0.01) and lower for young women (81% [IQR 51–98%]; *p* = 0.10).

**Table 2.** Overall participant-level adherence for the cohort and key subgroups

Cohort/Subgroup	*N* participants	Median (IQR) (%)	Mean (SD) (%)	*p*-Value*
Total	886	88 (64–99	77 (28)	–
Young adults (age <25)	176	83 (58–96)	74 (28)	0.06
Women	309	91 (66–99)	78 (29)	0.01
Women age <25	79	81 (51–98)	71 (32)	0.10

IQR: Interquartile range; SD: standard deviation. *p-Values reflect comparison of each subgroup with the remainder of the cohort.

Risk for HIV acquisition was considered high, low and very low at 753 (23%), 2086 (64%) and 446 (14%) of participant-visits, respectively (Table 3). Sufficient adherence defined as an average of ≥4 doses/week was seen in 663 (88%) of participant-visits with high risk for HIV acquisition, 1725 (83%) of participant-visits with low risk and 278 (62%) of participant-visits with very low risk (*p* < 0.0001). Sufficient adherence defined as an average of ≥6 doses/week was seen in 564 (75%) of participant-visits with high risk for HIV acquisition, 1433 (69%) of participant-visits with low risk and 217 (49%) of participant-visits with very low risk (*p* < 0.0001). Additionally, PrEP was prescribed, but not accepted by the participant at 166 study visits (5%); risk for HIV acquisition was considered high, low and very low in 6 (4%), 75 (45%) and 85 (51%) of these participant-visits, respectively. Cross-tabulations for young adults, women and young women revealed similar results (see Appendix).

**Table 3.** Prevention-effective adherence

	Sufficient adherence				
		≥4 Doses/week		≥6 Doses/week	
Risk of HIV acquisition	*N* participant-visits	% Participant-visits	*p*-Value*	% Participant-visits	*p*-Value*
High	753	88 (663/753)	<0.0001	75 (564/753)	<0.0001
Low	2086	83 (1725/2086)		69 (1433/2086)	
Very low	446	62 (278/446)		49 (217/446)	
Total	3285	81 (2666/3285)	–	67 (2214/3285)	–

*The *p*-value indicates if the proportion of participant-visits with sufficient adherence varies by risk category. This table indicates the percentage of participant-visits in which adherence is sufficient for protection against HIV acquisition categorized by risk. Sufficient adherence is estimated at an average of ≥4 doses/week and ≥6 doses/week in a participant month. Categories of risk are mutually exclusive for a given estimate of sufficient adherence.

During the twelve-month study period, 325 (37%) of participants did not change risk categories: 43 with high risk, 265 with low risk and 17 with very low risk. Risk could only be assessed once for 88 participants (10%). For the remaining 473 participants (53%), categories of HIV risk changed at least once during the study.

### Factors associated with prevention-effective adherence

Univariable analysis results are presented in the Appendix. In multivariable analysis  (Table 4), an average adherence of ≥4 doses/week was associated with no reported concerns about taking daily PrEP (RR 1.26; *p* < 0.0001), pregnancy or pregnancy intention (RR 1.05–1.07; *p* = 0.01), no longer being a couple with the study partner (RR 0.63; *p* < 0.0001), delayed PrEP initiation (RR 0.56–0.71; *p* = 0.045) and follow-up for >6 months  (RR 0.94; *p* = 0.003). Females of all ages were less likely to achieve ≥4 doses/week (RR 0.89; *p* = 0.03); however, females >25 years  were more likely to do so (RR 1.15; *p* = 0.007). An average of ≥6 doses/week was associated with reported sex with the study partner (RR 1.54 [100% condom use], 1.62 [<100% condom use]; *p* = 0.008), no concerns about daily PrEP (RR 1.43; *p* < 0.0001), females >25 years (RR 1.32; *p* = 0.0002), wanting “very much” or “desperately” for the relationship to succeed (RR 1.22; *p* = 0.002), male partner >5 years  older than the female partner (RR 1.08; *p* = 0.02), no longer being a couple with the study partner (RR 0.63; *p* = 0.001), problem alcohol use (RR 0.90; *p* = 0.01), follow-up >6 months  (RR 0.88; *p* < 0.0001) and ART use >6 months  by the partner living with HIV (RR 0.92; *p* = 0.03).

**Table 4.** Multivariable regressions of factors associated with sufficient adherence for protection against HIV infection, defined as ≥4 doses/week and ≥6 doses/week

	≥4 Doses/week			≥6 Doses/week		
Predictor	Prevalence withsufficient adherence (%)	RR (95% CI)	*p*-Value	Prevalence withsufficient adherence (%)	RR (95% CI)	*p*-Value
**HIV-uninfected partner enrolment characteristics**						
Female	82	**0.89 (0.79–0.99)**	**0.03**	71	0.88 (0.73–1.06)	0.17
Age ≥twenty five (years) and female*	84	**1.15 (1.04–1.27)**	**0.007**	75	**1.32 (1.14–1.54)**	**0.0002**
Age ≥twenty-five (years) and male	79	0.96 (0.89–1.03)	0.24	65	1.03 (0.90–1.17)	0.69
Married to study partner	80	–	–	67	1.12 (0.95–1.32)	0.19
Male partner is ≥five years older than female	82	–	–	69	**1.08 (1.01–1.16)**	**0.02**
**HIV risk factor time-varying characteristics**						
Any sex (study or other partner) in past 30 days	83	1.03 (0.83–1.28)	0.81	69	0.75 (0.53–1.07)	0.11
Sex with study partner in past 30 days No sex Sex, 100% condom use Sex, <100% condom use	62 83 86	ref 1.14 (0.93–1.40) 1.17 (0.95–1.44)	0.17	49 69 73	**ref** **1.54 (1.09–2.16)** **1.62 (1.14–2.29)**	**0.008**
ART use ≥six months by partner living with HIV	76	0.95 (0.91–1.01)	0.08	61	**0.92 (0.85–0.99)**	**0.03**
**Other HIV-uninfected partner time-varying characteristics**						
No concerns for taking daily PrEP	82	**1.26 (1.12–1.41)**	**<0.0001**	68	**1.43 (1.22–1.67)**	**<0.0001**
Wants relationship to succeed “very much”/ ’desperately“ (vs. “would be nice”/“never can”)	83	1.09 (1.00–1.19)	0.06	70	**1.22 (1.08–1.39)**	**0.002**
Pregnancy intentions Not pregnant, not trying Not pregnant, trying Currently pregnant	80 89 86	**ref** **1.07 (1.01–1.12)** **1.05 (1.00–1.11)**	**0.01**	66 76 70	ref 1.08 (0.99–1.18) 1.05 (0.97–1.14)	0.15
In follow-up >6 study months	75	**0.94 (0.90–0.98)**	**0.003**	59	**0.88 (0.82–0.94)**	**<0.0001**
No longer being a couple with study partner	47	**0.63 (0.51–0.79)**	**<0.0001**	34	**0.63 (0.48–0.83)**	**0.001**
Study month of PrEP initiation Baseline Month 1 >Month 1	81 44 50	**ref** **0.56 (0.36–0.88)** **0.71 (0.34–1.48)**	**0.045**	67 29 50	ref 0.49 (0.26–0.92) 0.94 (0.44–2.00)	0.07
Problem alcohol use	78	0.95 (0.90–1.01)	0.10	62	**0.90 (0.82–0.97)**	**0.01**
As much social support as desired	79	0.96 (0.92–1.01)	0.10	–	–	–

Statistical significance is considered at *p* < 0.05 (bold). “–” indicates predictor not included in the model under the specified definition of sufficient adherence. Predictors found not to be associated at *p* ≤ 0.10 on univariable analyses: education, living with study partner, in a polygamous marriage, no children with study partner, aware of HIV discordance at enrolment, CD4 cell count, viral load, circumcision (HIV-uninfected males only), any unprotected sex with a non-study partner, perceived HIV risk, perceived PrEP efficacy, effect of discordance on the relationship, relationship satisfaction, relationship happiness, abuse (verbal, physical or economic), depression and perceived stigma. No factors retained in the multivariable model were collinear. *Interaction between age and gender significant at 0.003 for the first model (≥4 doses/week) and 0.01 for the second model (≥6 doses/week).

## Discussion

In this demonstration project involving a scalable, integrated and pragmatic delivery approach with time-limited PrEP during the first six months of ART use, adherence sufficient to protect against HIV acquisition was achieved for 75–88% of participant-visits with high risk. Although we made assumptions about the number of doses/week sufficient to protect against HIV acquisition, these levels of adherence were associated with the low HIV incidence (0.2 infections/100 person-years) found in the Partners Demonstration Project [30]. Consistent with our findings, other open-label studies published since the efficacy of PrEP was determined in early clinical trials [1], including the Demo Project [7], ADAPT [8], IPERGAY [31] and PROUD [32], have found high levels of adherence and low incidence of HIV infection. The Demo Project similarly found that adherence was higher among those with higher reported risk for HIV acquisition (i.e. condomless receptive anal sex) [7].

Counselling messages in the Partners Demonstration Project did not specifically address the concept of prevention-effective adherence. Rather, HIV-uninfected participants who chose to take PrEP were advised to take PrEP daily, and PrEP was recommended to all couples until the partner living with HIV received ART for >6 months. Risk for HIV acquisition and other methods of protection were also discussed, including condoms, ART for individuals living with HIV, male circumcision and reduced numbers of sexual partners. The alignment of HIV risk and adherence presented in this analysis reflects the behaviour exhibited by the participants. As PrEP is used more frequently in clinical settings, counselling messages should be geared towards helping individuals understand and accurately assess their risk and choice among HIV prevention tools. Self-assessment of risk for HIV acquisition can be difficult [33], as it requires understanding of the mechanisms for HIV transmission, awareness of one’s own behaviour and sufficient information about the behaviour of one’s sexual partners. Despite those challenges, PrEP adherence in this demonstration project was statistically significantly higher during periods of sex with a potentially viremic partner, suggesting that individuals adhered better when they felt they needed protection against HIV infection. The similarity of results regardless of condom use may reflect challenges with achieving 100% condom use or misreporting of condom use due to social desirability bias.

The results of the regression analyses suggested specific motivators for PrEP use that may be helpful in supporting PrEP adherence and in identifying individuals for whom PrEP may be a good prevention option. For instance, individuals had higher adherence when they “very much” or “desperately” wanted their serodiscordant relationship to succeed; those pregnant or trying to get pregnant also had higher adherence. These factors were similarly found to be important for adherence among serodiscordant couples in the Partners PrEP Study [19,34]. PrEP for these participants was a means to relationship success and/or an HIV-uninfected baby. These desires may serve as better components of counselling messages than simply emphasizing the relationship between efficacy and adherence. Other studies have called for novel messaging approaches such as these to improve PrEP uptake [35,36]. Conversely, the lower adherence seen when the couple was no longer together further supports that PrEP may only make sense during certain times in people’s lives. Assessing an individual’s concern about taking daily PrEP may also help identify those for whom PrEP is most feasible. Lower adherence with ART use >6 months by the partner living with HIV is consistent with the message that PrEP is only needed for a time-limited period when used as a “bridge to ART”.

Despite these encouraging findings, as many as 25% of participant-visits indicated high risk for HIV infection in the setting of insufficient adherence. These periods suggest either the need for PrEP adherence interventions or potentially guidance for effective use of other HIV prevention tools (e.g. condoms). The regression analyses identified potential triggers for additional adherence support, including prolonged PrEP use (i.e. >six months) and problem alcohol use. The association of lower adherence with young women (i.e. age <25 years) suggests support is also needed for this population. However, it is important to note that median adherence was 81% – similar to the 76% adherence seen among women taking daily PrEP in ADAPT [8] and much higher than the <30% adherence seen in FEM-PrEP and VOICE [23,24].

The minority of participant-visits in which there was sufficient adherence but very low risk for HIV acquisition indicates some potentially unnecessary PrEP use, although the definition of risk used in this analysis was not comprehensive. For instance, HIV status in outside sexual partnerships was unknown. Importantly, risk can change quickly and some months with low risk in this analysis may have been flanked by months with higher risk. Starting and stopping PrEP on a frequent basis would be challenging, as at least one week of consistent use is likely needed to achieve protective tenofovir levels [10]. Rather, PrEP use should be considered as seasons (i.e. multiple months at a time) – another important feature for ongoing PrEP counselling.

This analysis has limitations. First, the definitions used for sufficient adherence and HIV risk may not be accurate for all individuals. Wide inter- and intra-individual variability exists for tenofovir levels and not all individuals will achieve the same level of protection with the same dosing [37]. We also do not have tenofovir pharmacokinetic data in penile tissue to make specific recommendations anything less than daily dosing. However, there is indirect evidence that suggests the protective dose frequency is similar to women – namely, the closer histologic similarity of penile compared to vaginal tissue in contrast to colorectal tissue, similar seroconversion rates between heterosexual men and women in Partners PrEP and TDF2 trials, and the excellent concentration-response relationship in those studies without adjustment for tissue pharmacokinetics [38]. Second, sufficient adherence was calculated in terms of doses per week averaged over the month prior to the participant-visit when sexual behaviour was reported, not per-sexual exposure. Recall bias and/or social desirability may therefore limit the accuracy of these assessments. We also did not factor in the need for several daily doses upon PrEP initiation to achieve the steady-state tenofovir levels necessary for HIV protection. Third, electronic adherence measurements used in this study may not have always been accurate. Individuals may have taken out >1 pill with each opening (i.e. pocket doses) or opened the cap without removing a pill (i.e. curiosity openings) or ingesting it (i.e. device manipulation). Finally, this analysis was limited to individuals with concurrent electronic adherence data and reported sexual risk behaviour, which comprised approximately 70% of all participant-visits in the study. Adherence would clearly be lower when including participants who chose not to take it. However, the goal of this analysis was to assess the alignment of adherence and HIV risk in those choosing to take PrEP. The impact of missing data for adherence or risk behaviours on our findings is unknown.

In conclusion, this study adds to the growing evidence that most individuals adhere to PrEP sufficiently for effective HIV prevention most of the time – a strong argument for expanding access to PrEP. This study also presents a novel analytic approach to aligning adherence with risk for HIV acquisition. Future efforts should explore prevention-effective adherence in different populations and cultural contexts, as well as develop counselling messages for routine clinical delivery of PrEP and other HIV prevention tools that help individuals understand their risk and assist in aligning adherence with risk. SMS could be a useful tool for ongoing HIV risk assessment and could be paired with real-time adherence monitoring to refine and support prevention-effective adherence [39,40]. Optimal alignment of HIV prevention tools with risk will help increase the effectiveness of HIV prevention efforts, thus maximizing individual and public health benefits.

## Partners Demonstration Project Team

Coordinating Center (University of Washington) and collaborating investigators (Harvard Medical School, Johns Hopkins University, Massachusetts General Hospital): Jared Baeten (protocol chair), Connie Celum (protocol co-chair), Renee Heffron (project director), Deborah Donnell (statistician), Ruanne Barnabas, Jessica Haberer, Harald Haugen, Craig Hendrix, Lara Kidoguchi, Mark Marzinke, Susan Morrison, Jennifer Morton, Norma Ware, Monique Wyatt.

## Project sites

Kabwohe, Uganda (Kabwohe Clinical Research Centre): Stephen Asiimwe, Edna Tindimwebwa

Kampala, Uganda (Makerere University): Elly Katabira, Nulu Bulya

Kisumu, Kenya (Kenya Medical Research Institute): Elizabeth Bukusi, Josephine Odoyo

Thika, Kenya (Kenya Medical Research Institute, University of Washington): Nelly Rwamba Mugo, Kenneth Ngure

Data Management was provided by DF/Net Research, Inc. (Seattle, WA). PrEP medication was donated by Gilead Sciences.

## Supplemental Material

To access the supplementary material to this article please see Supplementary Files under Article Tools online.
